# Utility of a Work Process Classification System for characterizing non-fatal injuries in the Alaskan commercial fishing industry

**DOI:** 10.3402/ijch.v75.30070

**Published:** 2016-01-14

**Authors:** Laura N. Syron, Devin L. Lucas, Viktor E. Bovbjerg, Jeffrey W. Bethel, Laurel D. Kincl

**Affiliations:** 1 School of Biological and Population Health Sciences, College of Public Health and Human Sciences, Oregon State University, Corvallis, OR, USA; 2 National Institute for Occupational Safety and Health, Western States Division, Anchorage, AK, USA

**Keywords:** occupational safety, non-fatal injuries, commercial fishing, Alaska, work process

## Abstract

**Background:**

The US commercial fishing industry is hazardous, as measured by mortality data. However, research on non-fatal injuries is limited. Non-fatal injuries constitute the majority of occupational injuries and can result in workers’ lowered productivity and wages, lost quality of life, and disability. In the United States, a Work Process Classification System (WPCS) has previously been applied in Alaskan freezer-trawl and freezer-longline fleets to identify causes of injuries and specific hazards, but not to other fishing fleets.

**Objectives:**

This descriptive epidemiologic study aimed to explore the application and modification of the WPCS in multiple Alaskan fleets, characterize non-fatal occupational injuries in these fleets, and identify work processes that could be targeted for further investigation and future injury prevention efforts.

**Design:**

Traumatic, non-fatal injuries on-board Alaskan commercial fishing vessels were identified through United States Coast Guard investigative reports. Characteristics of injuries, as well as worker characteristics, were analysed. Injuries were coded using the WPCS.

**Results:**

We successfully utilized the WPCS to code non-fatal injury cases (n = 136). The most frequent main work processes associated with non-fatal injuries included: on-board trawlers, *handling frozen fish* and *processing the catch*; on-board vessels using pot/trap gear, *handling the gear* and *shooting/setting the gear*; on-board longliners, *traffic on board* and *hauling the gear*; and on-board processor vessels, *processing the catch*, *other work with the catch*, and *handling frozen fish*.

**Conclusions:**

The study confirmed that a WPCS can be applied to multiple Alaskan fleets to identify hazardous tasks. Hazards were unique for each vessel gear type. Future injury prevention efforts should target work processes associated with the most frequent and most severe injuries. Future studies should establish time estimates for work processes in order to determine risk estimates. Efforts to improve non-fatal injury reporting, especially on smaller commercial fishing vessels, should be undertaken.

Commercial fishing is prevalent in the circumpolar region, including Alaska. It is one of the most hazardous industries in the United States, measured by mortality data. Data from the US Bureau of Labor Statistics (BLS) showed that in 2012 the fatal injury rate for fishers and related fishing workers (Standard Occupational Classification 45-3011) was 120.8 per 100,000 full-time equivalent workers, which was 35 times higher than the all-worker fatal injury rate of 3.4 per 100,000 full-time equivalent workers for that year (1). Given the hazardous nature of commercial fishing, research on occupational injuries in this industry is crucial to understanding hazards and preventing injuries. Non-fatal injuries (in contrast to fatal injuries) constitute the vast majority of occupational injuries and can result in workers’ lowered productivity, lost wages, lost quality of life, and disability. However, research on non-fatal injuries in the commercial fishing industry is limited.

In 2007, the National Institute for Occupational Safety and Health (NIOSH) created the Commercial Fishing Incident Database (CFID) to facilitate the collection of fatality data and identify high-risk fisheries in the United States. A review of commercial fishery fatality data in the CFID showed that 504 commercial fishing deaths occurred during 2000–2009 (2). Although the CFID captures data on fatal injuries in the commercial fishing industry, there is a dearth of information on non-fatal injuries.

The BLS Survey of Occupational Injuries and Illnesses (SOII) contains non-fatal occupational injury and illness data for all industries. However, it does not include data on self-employed workers. This is a major limitation of the BLS SOII for assessing the extent of non-fatal injuries in the commercial fishing industry, given that the vast majority of fishermen are self-employed. According to the BLS SOII, during 2003–2009 there were 610 non-fatal injuries and illnesses in fishing-related occupations across the nation (3). Results from the few studies that have addressed non-fatal injuries in the commercial fishing industry in various regions across the country demonstrate that these BLS national-level statistics have extremely underestimated the number of non-fatal injuries (4–9).

Companies that operate fishing vessels are required to report to the United States Coast Guard (USCG) any “injury that requires professional medical treatment (treatment beyond first aid) and, if the person is engaged or employed on board a vessel in commercial service, that renders the individual unfit to perform his or her routine duties” (Code of Federal Regulations, Title 46, Section 4.05 – 1). The CG-2692 *Report of Marine Casualty* form is used to report the details of the incident (10). The Coast Guard conducts investigations of injuries on fishing vessels that meet the reporting requirement and prepares reports on the findings. The full reports are not publicly available.

Lucas et al. (9) studied fatal and non-fatal injuries on-board freezer-trawlers and freezer-longliners operating in Alaska during 2001–2012. They utilized data from 2 sources: USCG investigation reports and reports of injuries filed by fishery observers posted on the vessels by the National Marine Fisheries Service (NMFS). Their study was the first to utilize a Work Process Classification System (WPCS) for fisheries in the United States. Originally, Jensen et al. (11–13) developed and pilot tested the WPCS in Danish fisheries. The purpose of the classification system was to better identify the causes of injuries and use it to effectively identify specific hazards in those fisheries. The original classification system contained 18 main work processes and 13 sub-processes for each fishing method, including Danish seiner, gill-netter, beam trawler, twin-trawler, and single/pair trawler. Although Danish and US fisheries differ considerably, Lucas et al. found that all 18 main work processes were applicable to the vessels that were included in the US study. However, many of the sub-processes were needed to be modified or replaced in order to properly categorize unique fishing procedures. The study found that during 2001–2012, workers in the freezer-trawler fleet experienced 384 non-fatal injuries, and workers in the freezer-longliner fleet experienced 294 non-fatal injuries. The authors concluded that using the WPCS, in conjunction with Occupational Injury and Illness Classification System (OIICS) (14) coding for the nature of injury and the injured body part, was an effective method for identifying the specific circumstances producing the most injuries in the fleets studied. The study was restricted to 2 particular Alaskan fleets of large factory vessels that used longline and trawl gear, and did not cover many other, smaller Alaskan fleets that utilize other gear types.

## Objective

Building on the research conducted by Lucas et al. on freezer-trawlers and freezer-longliners, the current study used a similar approach to analysing non-fatal traumatic injuries in additional Alaskan fishing fleets. Commercial fishing fleets are groups of vessels that focus on a certain species and/or operate out of the same port, region, or country. Different fleets may utilize the same general type of gear; for instance, crab, shrimp, and Pacific cod fleets can all use pot gear to harvest the catch (albeit different kinds of pots). The objectives of this descriptive epidemiologic study were to explore the application and modification of the WPCS in multiple Alaskan fleets, characterize non-fatal occupational injuries in these fleets, and identify work processes that could be targeted for further investigation and future injury prevention efforts. Given that hazards can vary according to fishing vessels’ specific gear types, injury prevention efforts can be tailored according to the gear type that is utilized.

## Design

### Case definition

Cases included non-fatal traumatic injuries to commercial fishing workers on-board vessels operating in Alaska during 2006–2010. The BLS describes traumatic injury as a “wound or damage to the body resulting from acute exposure to energy […] caused by a specific event or incident within a single workday or shift” (15). Injuries to NMFS fishery observers, who monitor fishery management rules, and collect catch and bycatch data while on-board commercial fishing vessels (16), were excluded from the study as they were not engaged in commercial fishing work.

### Data source

In collaboration with the NIOSH, Western States Division, Alaska Office, a USCG member collected information on non-fatal injuries that occurred on-board Alaskan vessels during 2006–2010. This data collection process 1involved reviewing USCG investigation reports, abstracting information from the reports, coding the relevant injury data, and entering the data into a study database. Through a partnership with NIOSH, our research team gained access to the data set for this study. This study was reviewed by the Oregon State University Institutional Review Board and determined to be exempt from full board review, as data were de-identified (study number 6386).

### Measures

Information about the worker, injury, circumstances surrounding the injury, and vessel were collected for each incident. Injury measures were coded (described below) to allow for detailed and standardized analysis. Worker characteristics included age, sex, years of experience in the commercial fishing industry, and position on board (captain, mate, deckhand, etc.). The nature of injury and the body part injured were coded using the OIICS (14). Level of care was reported as treatment on-board by crew member, at a clinic, or at a hospital. An adaptation of the Abbreviated Injury Scale that is used by USCG investigators was used to code injury severity, ranging from minor to critical (17).

NIOSH's CFID utilizes a WPCS that is modified for US fleets. This WPCS was originally developed by Jensen et al. in Danish fisheries (11–13). Lucas et al. (9) modified Jensen et al.'s system for use in the Alaskan freezer-trawl and freezer-longline fleets. As NIOSH reviews US commercial fishing incident data (including fatal injury data), it may at times identify work processes that are not currently included in the WPCS and then update the work process codes accordingly. In CFID's WPCS, the main work process codes include: *traffic at home and port*; *traffic on board*; *watch*; *preparing fishing gear*; *shooting/setting the gear*; *hauling the gear*; *handling the gear on deck*; *processing the catch*; *other work with the catch*; *handling frozen fish*; *preparing deck gear*; *working in engine room*; *mooring*; *working in the galley*; *off duty*; *other*; *diving*; and *unclassifiable*. The main work process codes that involve fishing gear (*preparing fishing gear*, *shooting/setting the gear*, *hauling the gear*, *handling the gear on deck*, and *preparing deck gear*) are further organized by vessels’ specific gear type: pots/traps; longliner; trawler; dredge; seiner; gillnet; troller; and other. On commercial fishing vessels, different gear types present unique hazards to workers and therefore injury prevention efforts can be tailored for specific gear types. Each main work process contains sub-processes that provide a more detailed level of description. Providing such detailed injury data by fishery and gear type is valuable for future injury investigation and prevention efforts.

In order to manually assign a work process code to each injury case in this study, the research team reviewed the following variables for each case: workers’ location on the vessel at the time of injury; workers’ position (deckhand, processor, etc.); event or exposure resulting in injury; primary cause of injury; contributing factor to injury; vessel activity; vessel type (catcher, processor, tender, dive, etc.); gear type; and a narrative description of the circumstances surrounding the injury. Work process coding was validated through a standard quality control process involving multiple research team members independently coding difficult cases and resolving differences. First, the lead author (LS) reviewed the applicable variables for each injury case. While coding the work process for each case, the lead author assigned that code with a confidence rating from 0 to 2, with 0 signifying that there was no confidence in the code's accuracy and 2 signifying absolute confidence in the code's accuracy. Cases assigned a confidence rating of 0 or 1 triggered a second, independent review by a co-author (LK). After the second co-author's independent review and work processing coding, unresolved codes and those with continued low-confidence ratings triggered a third independent review by another co-author (DL). As an expert in the field of commercial fishing safety research, the third co-author resolved any remaining uncertainties in the coding. During the coding process, if the research team could not identify an appropriate work process code that reflected the narrative description of the circumstances surrounding the injury then they generated a new code based on the non-fatal injury data.

### Analysis

Characteristics and causes of non-fatal injuries, as well as worker characteristics, were analysed. Descriptive statistics, including frequency and percent distributions, measures of central tendency and dispersion, and cross-tabulations were calculated for all non-fatal injuries, both in aggregate and by specific gear type.

## Results

A total of 141 injury cases on fishing vessels in Alaska were investigated by the USCG during 2006–2010. Of these, 136 cases met the inclusion criteria for this study as non-fatal traumatic injuries to commercial fishing workers on-board vessels operating in Alaskan waters.

### Worker and injury characteristics

Age was reported for 122 (90%) cases, with a median age of 35 years and range of 15–70 years. Sex was reported for 133 (98%) cases and all workers were male. Position on board was reported for 134 cases (99%), with 9 position categories identified among them. Processors (57, 42%) and deckhands (49, 36%) were the most frequently injured workers, followed by: “other” (10, 8%); engineer (6, 4%); captain (5, 4%), mate (3, 2%); baiter (2, 2%); cook (1, 1%), and diver (1, 1%). Workers’ years of experience in the industry was reported for 116 (85%) cases. The median number of years of experience was 4.5 years, with a range of 0–45 years of experience. The year that the injury occurred was reported for all cases: 2006 (29, 21%), 2007 (50, 37%), 2008 (15, 11%), 2009 (30, 22%), and 2010 (12, 9%).

The body part injured and the nature of injury were reported for all cases. Injuries to the upper extremities (48, 35%) and fractures (36, 26%) were most frequently reported (Table I).

**Table I:** Nature and body part of all non-fatal injuries investigated by United States Coast Guard in Alaska, 2006–2010

Nature of injury	Body part injured No. (% total)					

No. (% total)	Upper extremities	Trunk	Lower extremities	Head	Multiple	Total
Fracture	14	7	13	1	1	36 (26)
Lacerations, puncture	10	1	1	11	0	23 (17)
Sprains, strains, tears	0	11	8	0	0	19 (14)
Amputations	13	0	2	0	0	15 (11)
Bruises, contusions	3	4	1	1	0	9 (7)
Intracranial injuries	0	0	0	9	0	9 (7)
Burns	4	1	0	1	0	6 (4)
Hernia	0	3	0	0	0	3 (2)
Multiple	0	2	0	0	1	3 (2)
Dislocation	0	2	0	0	0	2 (1)
Environmental conditions	0	1	0	0	1	2 (1)
Unspecified	4	1	3	1	0	9 (7)
Total	48 (35)	33 (24)	28 (21)	24 (18)	3 (2)	136 (100)

Injury severity was reported for all cases: “critical” (3, 2%), “severe” (8, 6%); “serious” (31, 23%), “moderate” (68, 50%); and “minor” (26, 19%). The level of care that a worker received for an injury was reported for 126 cases (93%). The most frequent level of care was being seen at a clinic (64, 51%), followed by seeking treatment at a hospital (43, 34%) and being treated by a crewmember on board the vessel (19, 15%).

### Work processes

All 136 cases in the study were coded with work process classifications. Three cases were coded as unclassifiable due to lack of information on what the injured worker was doing at the time of the injury, with 2 of these cases involving assault. Thirteen main work processes were identified. The most frequent main work processes associated with injuries were *traffic on board* (25, 18%), *handling frozen fish* (25, 18%), *processing the catch* (21, 15%), and *hauling the gear* (16, 12%). For both *traffic on board* and *handling frozen fish* the most common nature of injuries were sprains/strains/tears and fractures (Table II). The Supplementary file presents the frequency and percent of work processes for all cases at the most detailed sub-process level and indicates the 6 sub-process codes that were revised or created during this study.

**Table II:** Main work process and nature of all non-fatal injuries investigated by United States Coast Guard in Alaska, 2006–2010

	Nature of Injury												
	
Main work Process	Fracture	Laceration/puncture	Sprain/ strain/tear	Amputation	Contusion	Intracranial Injury	Burn	Hernia	Multiple	Dislocation	Environ. Condition	Unspecified	Total
Traffic on board	6	2	7	0	2	3	0	0	1	1	0	3	25
Shooting/setting the gear	2	0	0	0	1	1	0	0	1	0	0	0	5
Hauling the gear	5	2	1	4	1	1	0	0	0	1	0	1	16
Handling frozen fish	6	1	7	1	2	2	3	1	0	0	0	2	25
Handling the gear	5	2	0	1	0	0	0	0	1	0	0	1	10
Processing the catch	5	6	2	5	2	0	0	0	0	0	0	1	21
Other work with the catch	2	0	0	0	0	2	0	0	0	0	0	0	4
Working in engine room	0	0	0	1	0	0	1	0	0	0	0	0	2
Mooring	1	0	1	1	0	0	0	0	0	0	0	0	3
Off duty	1	4	0	0	0	0	0	0	0	0	0	1	6
Other	2	4	1	1	1	0	2	2	0	0	1	0	14
Diving	0	0	0	1	0	0	0	0	0	0	1	0	2
Unclassifiable	1	2	0	0	0	0	0	0	0	0	0	0	3
Total	36	23	19	15	9	9	6	3	3	2	2	9	136

### Results by gear type

Of the 136 cases, information on vessel gear type was available for 129 (95%) cases. Of these cases, the number and percentage of cases by gear types included: dive gear (1, 1%); gillnet (3, 2%); seine (5, 4%); longline (15, 12%); pot/trap (19, 15%); trawl (69, 53%); and no fishing gear on the vessel, i.e. processor and tender vessels (17, 13%). Brief descriptions of each gear type are provided below. Alaskan fleets have been described in detail in the Alaska Sea Grant College Program's publication *Ocean Treasure: Commercial Fishing in Alaska* by Johnson (18).

### Dive, gillnet, and seine gear

Dive gear is used to harvest urchins, sea cucumbers, and geoducks (18). One case occurred on a vessel with dive gear. The injury severity was moderate and the work process was *diving*.

Gillnetting, utilizing a net made of lightweight twine woven into panels of mesh, is a method used to harvest salmon and herring (18). Three cases occurred on vessels with gillnet gear. Of the 3 vessels on which injuries occurred, 2 were using drift gillnet gear. The 2 injuries were of minor and moderate severity. The main work processes included *fighting fire* and *diving, non-specified*. The third vessel on which an injury occurred was a set gillnet skiff and was severe. In this instance, the work process was *off duty* – *sleeping*.

Seining is a method used to harvest salmon and herring. It involves using a curtain-like net to scoop under a school of fish, usually within a mile of shore (18). Five cases occurred on vessels with seine gear. Two intentional injuries that resulted from assault were coded with the main work process *off duty*. Of the 3 unintentional injuries, 2 occurred during *hauling the gear* and 1 occurred during *working in engine room*. Across all cases, injury severity ranged from minor to critical. The case with the worst injury severity category of critical occurred during *hauling the gear*.

### Longline gear

Longlining with baited hooks is a method for harvesting halibut, sablefish, rockfish, and Pacific cod (18). Fifteen cases occurred on vessels with longline gear. The most frequent main work process was *traffic on board* (7, 47%). Approximately half of the injuries were of moderate severity (8, 53%) (Table III).

**Table III:** Work process and severity of non-fatal injuries on-board longliners investigated by United States Coast Guard in Alaska, 2006–2010

	Severity of injury				
	
Work process	Minor	Moderate	Serious	Severe/critical	Total
Traffic on board					
Traffic on deck	1	3	1	0	5
Traffic in freezer	0	0	1	0	1
Traffic on ladders/stairs	0	0	0	1	1
Hauling the gear					
Running the longline roller	0	1	0	0	1
Pulling up the flagpole/buoy/anchor	0	1	0	0	1
Processing the catch					
Lifting fish onto table	0	1	0	0	1
Handling frozen fish	0	0	1	0	1
Mooring	0	0	1	0	1
Other					
Shovelling snow/breaking ice	0	1	0	0	1
Fighting fire	1	0	0	0	1
Unclassifiable	0	1	0	0	1
Total	2	8	4	1	15

### Pot/trap gear

Pot/trap gear is used to catch crab, shrimp, and Pacific cod. Pots have a welded steel frame that is covered with polyester or stainless steel mesh. The size and style vary depending on the area being fished and the species being caught (18). Nineteen cases occurred on vessels with pot/trap gear. The most frequent main work processes were *handling gear on deck* (8, 42%), *shooting/setting the gear* (4, 21%), and *hauling the gear* (3, 16%). The majority of injury severity categories were “moderate” (11, 58%), followed by “serious” (5, 26%) (Table IV).

**Table IV:** Work process and severity of non-fatal injuries on-board pot/trap gear vessels investigated by United States Coast Guard in Alaska, 2006–2010

	Severity of injury				
	
Work process	Minor	Moderate	Serious	Severe/critical	Total
Shooting/setting the gear					
Throwing pots	0	1	0	0	1
Operating pot launcher	0	1	1	0	2
Untying pots	0	0	1	0	1
Hauling the gear					
Operating the pot/trap hauler	0	1	0	0	1
Pushing pot to stack	0	1	0	0	1
Hauling the pot/trap gear	0	0	1	0	1
Handling the gear					
Securing gear, non-specified	0	3	0	0	3
Operating crane	0	1	0	0	1
Working pot stacks	0	0	1	1	2
Handling pot/trap gear on deck	1	1	0	0	2
Processing the catch					
Counting and sorting fish/crab	0	1	0	0	1
Processing the catch	0	0	1	0	1
Mooring					
Handling lines during mooring	1	0	0	0	1
Off duty					
Taking shower	0	1	0	0	1
Total	2	11	5	1	19

### Trawl gear

Trawlers tow nets to harvest cod, Pollock, sole, and other groundfish. They are large vessels, ranging from 60 to over 300 feet. They can be catcher vessels that harvest fish, or factory trawlers (known as catcher-processors) that catch, process, and freeze fish (18). Half of the cases in this study, 69 cases, occurred on vessels that used trawl gear. Eight main work processes were identified. Most injuries occurred when *handling frozen fish* (22, 32%), *processing the catch* (15, 22%), *traffic on board* (13, 19%), *hauling the gear* (8, 12%), and *other* (8, 12%). Across all cases, injury severity ranged from minor to severe. The most frequent injury severity category was moderate (37, 54%) (Table V).

**Table V:** Work process and severity of non-fatal injuries on-board trawlers investigated by United States Coast Guard in Alaska, 2006–2010

	Severity of injury				
	
Work process code	Minor	Moderate	Serious	Severe/critical	Total
Traffic on board					
Traffic on deck	0	3	2	1	6
Traffic in cabin/galley/bunk	0	0	1	0	1
Traffic in factory	0	2	0	0	2
Traffic in freezer	0	0	1	0	1
Traffic on ladders/stairs	0	1	1	1	3
Hauling the gear					
Pulling on slack trawl wire	0	0	1	0	1
Pushing fish from trawl deck into hold	0	1	0	1	2
Pulling up net	0	3	1	0	4
Hauling the trawl gear	0	1	0	0	1
Handling the gear					
Handling trawl gear on deck	0	0	1	0	1
Processing the catch					
Gutting the catch	0	1	0	0	1
Bleeding the fish	1	0	0	0	1
Packing fish in pans	1	0	0	0	1
Heading the catch	1	1	1	0	3
Skinning fish	0	1	0	0	1
Processing the catch	2	4	1	1	8
Handling frozen fish					
Stacking blocks/bags of fish/crab	1	2	1	0	4
Remove fish from conveyor belt	1	0	0	0	1
In freezer offloading product	1	2	0	0	3
Bagging/casing blocks of fish/crab	0	1	0	0	1
Loading plate/blast freezers	0	4	0	0	4
Unloading plate/blast freezers	0	1	0	0	1
Handling frozen fish	3	4	1	0	8
Working in engine room	0	0	1	0	1
Off duty					
In bunk/stateroom	0	0	1	0	1
Other					
General maintenance work	0	2	0	0	2
Getting the ship ready for sea	0	1	0	0	1
Repair/Maintaining refrigeration system	0	1	0	0	1
Repairing conveyor motor	0	1	0	0	1
General vessel repair	0	0	1	0	1
Repair/maintaining processing machinery	1	0	0	1	2
Total	12	37	15	5	69

### No fishing gear: tender or processor vessels

Seventeen cases occurred on vessels that did not have fishing gear. Of these cases, 7 occurred on tenders and 10 on processor vessels. Tender vessels transport the harvest from catcher vessels to shore, as well as transporting supplies to catcher vessels (18). Of the 7 cases on-board tender vessels, 3 injuries were intentional and the result of assault. The main work processes for these 3 injuries were *off duty* – *sleeping* (1 case) and *unclassifiable* (2 cases). The injury severity ranged from minor to serious. Four unintentional injuries occurred during *traffic on board* (1 case), *other work with the catch* (1 case), and *other* (2 cases).

Ten injuries occurred on-board processor vessels. Most injuries occurred during *processing the catch* (3, 30%), *other work with the catch* (3, 30%), and *handling frozen fish* (2, 20%). Injury severity across all cases ranged from minor to serious. Serious injuries occurred during *other work with the catch*, *handling frozen fish*, and *other – general maintenance work* (Table VI).

**Table VI:** Work process and severity of non-fatal injuries on-board processor vessels without gear investigated by United States Coast Guard in Alaska, 2006–2010

	Severity of injury				
	
Work process code	Minor	Moderate	Serious	Severe/critical	Total
Traffic on board					
Traffic on deck	0	1	0	0	1
Processing the catch					
Counting and sorting fish/crab	0	1	0	0	1
Processing the catch, unspecified	2	0	0	0	2
Other work with the catch					
Offloading fish	0	0	1	0	1
Loading brailer	0	1	0	0	1
Moving skate roller	0	1	0	0	1
Handling frozen fish					
Cracking pans	1	0	0	0	1
Handling frozen fish	0	0	1	0	1
Other					
General maintenance work	0	0	1	0	1
Total	3	4	3	0	10

## Discussion

This study fills a gap in research on non-fatal occupational injuries in the Alaskan commercial fishing industry. By including fleets that use dive, gillnet, seine, and pot/trap gear, as well as tender and processor vessels, it expands on a previous study that only focused on injuries on-board freezer-trawler and freezer-longliner fleets (9). This study provides evidence that the WPCS (9, 11–13) can be used successfully in fleets utilizing these additional gear types. In order to manually code the work process associated with each non-fatal injury case, the research team reviewed the applicable variables and narratives for each case and completed a standard quality control procedure in order to validate coding accuracy. In this study, we defined “success” in using the WPCS as our ability to utilize variables and narratives from USCG reports to determine the work process associated with the non-fatal injury. We determined that the main work process codes were applicable to the non-fatal injury cases in additional fleets. Only 6 sub-process codes (which provide the highest level of detail) were created or revised during the study when an appropriate sub-process code was not already available in the WPCS. The creation of new sub-process codes assisted with populating NIOSH's CFID with work process codes based on non-fatal injury data. The research team experienced difficulty only when assigning a work process code to an injury case when the USCG reports provided insufficient detail about the injury circumstances. This type of limitation is true for any classification system relying on narratives in the data source. By publishing the codes used in this study, we hope to make using the WPCS for injury epidemiology research sustainable. The WPCS is likely suitable for broad application to fleets across the circumpolar region. Future studies should engage the fishing industry, including workers, to validate the current work process codes for each fishery and to identify additional work processes not yet captured in the WPCS.

The study's detailed results could be used as starting points for informing non-fatal commercial fishing injury research. As previously noted (9), the main work processes associated with the highest frequencies of injuries on-board trawlers included *handling frozen fish*, *processing the catch*, and *traffic on board*, and on-board longliners included *traffic on board*, *hauling the gear*, and *handling frozen fish*. This study identified that on-board vessels using pot/trap gear, the most frequent main work processes involved working with gear, including *handling the gear*, *shooting/setting the gear*, and *hauling the gear*. Unsurprisingly, the main work processes identified on-board processor vessels included *processing the catch*, *other work with the catch*, and *handling frozen fish*. This highlights the importance of analysing the more detailed sub-processes in order to create targeted injury prevention strategies by specific hazards in each fleet.

In certain instances, the work process codes alone do not provide sufficient descriptions of the circumstances surrounding injuries to be useful for developing prevention strategies. For example, this study identified 5 intentional injuries resulting from assault, which were coded with the main work processes *off duty* (3) and unclassifiable (2). For injuries that occur due to vessel disasters, work processes may not indicate the factors that would need to be modified in order to prevent future incidents. Therefore, work process results should be paired with information from other variables, including the intentionality of injuries and type of incident (on-board injury, fall overboard, vessel disaster, etc.). Additionally, the example of intentional injuries points to the dearth of studies addressing workplace violence in the commercial fishing industry, which is likely to require distinct prevention strategies.

Estimated time spent performing each work process – the denominator data which would be needed for determining the highest-risk work processes – were unavailable. This limitation has been noted by Jensen et al. (13) in a study on relating the length of working time to the number of injuries for specific work processes in the Danish fishing industry. The authors stated that risk assessment should be based on the numerical values of the injuries and/or the seriousness of the injuries, and that when time estimate data are available then they should be utilized as supplemental indicators of risk. This approach was taken in the current study by presenting detailed information on work processes and injury severity for specific vessel and gear types. Future studies should address timing each work process in US commercial fishing fleets.

Although the sample size for this study was small, there was a variety of fleets included to test the application of the WPCS. In other respects, the small sample size of 136 cases was a true limitation. It is likely that non-fatal injuries, especially those of lower severity, were underreported and therefore not investigated by the USCG, as has been noted in previous studies (7,9). Non-fatal injuries of serious (23%) and moderate (50%) severity were reported more frequently than those of minor (19%) severity. This is most likely because less severe injuries did not require medical treatment and therefore the USCG was either not contacted, or chose not to investigate the incidents. Additionally, during 2005–2007, the USCG increased its efforts to improve fishing companies’ reporting of injuries (9). This initiative could explain why the highest number of cases (50, 37%) was reported in 2007, suggesting that increased reporting rather than an increase in the actual number of non-fatal injuries experienced by commercial fishing workers occurred in 2007. Although roughly half of the cases in this study occurred on-board trawlers, the risk of non-fatal injury was not necessarily higher on-board trawlers, but rather non-fatal injuries were more likely to be reported to the USCG. Natural Resources Consultants, Inc. commercial fishing employment estimate data show that during 2006–2010, there was an annual average of 10,860 active Alaskan commercial fishing vessels of all types (pot/trap, longline, trawl, seine, gillnet, processor, tender). Of all these vessels, there was an annual average of only 350 trawlers, constituting 3% of all active vessels (19). These figures suggest that non-fatal injuries were grossly underreported on the other, smaller vessel types. Large trawlers are owned by large companies that have more corporate and government oversight and incentives for complying with the USCG reporting requirements.

## Conclusions

Utilizing a WPCS, this descriptive epidemiologic study analysed non-fatal injuries in the Alaska commercial fishing industry that were investigated by the USCG during 2006–2010. Given that hazards were unique across the various vessel gear types, non-fatal injury investigation efforts should target the work processes most frequently associated with injury, particularly severe injury. This study found that non-fatal injuries most frequently occurred during the following main work processes: on-board trawlers, *handling frozen fish*, *processing the catch*, and *traffic on board*; on-board vessels using pot/trap gear, main work processes that involve working with gear, including *handling the gear*, *shooting/setting the gear*, and *hauling the gear*; and on-board longliners, *traffic on board* and *hauling the gear*. Future studies that expand this study's longitudinal and geographical scope could better characterize work processes during which fishermen experience non-fatal injuries on-board different fleets. Additionally, future studies on commercial fishing safety could establish time estimates for work processes in order to determine risk estimates. Efforts to improve non-fatal injury reporting, especially on smaller commercial fishing vessels, should be undertaken.

## Supplementary Material

Utility of a Work Process Classification System for characterizing non-fatal injuries in the Alaskan commercial fishing industry
