# Supplementary material for: Utility of a Work Process Classification System for characterizing non-fatal injuries in the Alaskan commercial fishing industry
Source: Int J Circumpolar Health. 2016 Jan 14;75:10.3402/ijch.v75.30070. doi: 10.3402/ijch.v75.30070 (PMC4717151; doi:10.3402/ijch.v75.30070)
Supplement: Utility of a Work Process Classification System for characterizing non-fatal injuries in the Alaskan commercial fishing industry [file IJCH-75-30070-s001.pdf]

SUPPLEMENTARY MATERIAL

**SUPPLEMENTAL TABLE:**<br>Complete Work Process Codes for Non-Fatal Injuries Onboard All Alaskan Fleets, 2006 - 2010					
			No.	%	Revised or Created *
**2 Traffic on board**			**25**	**18.4**	
	200	Embarking/disembarking	2		
	201	Traffic on deck	14		
	202	Traffic in cabin/galley/bunk	1		
	204	Traffic in factory	2		
	205	Traffic in freezer	2		
	206	Traffic on ladders/stairs	4		
**5 Shooting/setting the gear**			**5**	**3.7**	
	50	Pots/traps			
	5000	Throwing pots	1		
	5003	Operating pot launcher	2		
	5004	Untying pots	1		
	59	Other or non-specified gear type			
	5999	Shooting other or non-specified gear type, non-specified	1		
**6 Hauling the gear**			**16**	**11.7**	
	60	Pots/traps			
	6000	Operating the pot/trap hauler	1		
	6008	Pushing pot to stack	1		*
	6099	Hauling the pot/trap gear,<br>non-specified	1		
	61	Longliner			
	6100	Running the longline roller	1		
	6103	Pulling up the flagpole/buoy/anchor	1		*
	62	Stern Trawler			
	6202	Pulling on slack trawl wire	1		
	6203	Pushing fish from trawl deck into hold	2		
	6205	Pulling up net	4		
	6299	Hauling the trawl gear, non-specified	1		
	65	Seine			
	6500	Hauling seine net into boat	1		
	6503	Skiff operations	1		
	69	Other or non-specified gear type			
	6999	Hauling other or non-specified gear type, non-specified	1		
**7 Handling the gear**			**10**	**7.4**	
	70	Pots/traps			
	7000	Securing gear, non-specified	3		
	7004	Operating crane, non-specified	1		
	7007	Working pot stacks, non-specified	2		
	7099	Handling pot/trap gear on deck, non-specified	2		
	72	Stern Trawler			
	7299	Handling trawl gear on deck, non-specified	2		
**8 Processing the catch**			**21**	**15.4**	
	800	Counting and sorting fish /crab	2		
	803	Lifting fish onto table	1		
	804	Gutting the catch	1		
	805	Bleeding the fish	1		
	808	Packing fish in pans	1		
	809	Heading the catch	3		
	815	Skinning fish	1		*
	899	Processing the catch, non-specified	11		
**9 Other work with the catch**			**4**	**2.9**	
	903	Offloading fish	2		
	905	Loading brailer	1		*
	906	Moving skate roller	1		*

**10 Handling frozen fish**			**25**	**18.4**	
	1000	Stacking blocks/bags of fish/crab	4		
	1001	Cracking pans	1		
	1002	Remove fish from conveyor belt	1		
	1004	In freezer offloading product	3		
	1005	Bagging/casing blocks of fish/crab	1		
	1007	Loading plate/blast freezers	4		
	1008	Unloading plate/blast freezers	1		
	1099	Handling frozen fish, non-specified	10		
**12 Working in engine room**			**2**	**1.5**	
	1200	Engine maintenance	1		
	1299	Working in engine room, non-specified	1		
**13 Mooring**			**3**	**2.2**	
	1300	Mooring	2		
	1301	Handling lines during mooring	1		
**15 Off Duty**			**6**	**4.4**	
	1500	Sleeping	2		
	1501	Eating	2		
	1502	Taking shower	1		
	1504	In bunk/stateroom	1		
**16 Other**			**14**	**10.3**	
	1601	General maintenance work	4		
	1602	Getting the ship ready for sea	1		
	1606	Repairing/Maintaining refrigeration system	1		
	1608	Shoveling snow/breaking ice	1		
	1609	Repairing conveyor motor	1		
	1613	Securing/Closing hatch	1		
	1614	General vessel repair	1		
	1615	Fighting fire	2		
	1616	Repairing/Maintaining processing machinery	2		*
**17 Diving**			**2**	**1.5**	
	1799	Diving, non-specified	2		
**9999 Unclassifiable**			**3**	**2.2**	
		Total	136	100	
